# Cost Estimation Analysis of Dementia: A Scope Review

**DOI:** 10.7759/cureus.84547

**Published:** 2025-05-21

**Authors:** Stella Kalochristianaki, Pinelopi Vlotinou, George Bablekos, Ioanna Giannoula Katsouri, Anna Tsiakiri, Vasiliki Georgousopoulou, Georgia Tsakni

**Affiliations:** 1 Occupational Therapy, Health Canada, Athens, GRC; 2 Occupational Therapy/Neurological Rehabilitation, University of West Attica, Athens, GRC; 3 Occupational Therapy, University of West Attica, Athens, GRC; 4 Medicine, Democritus University of Thrace, Alexandroupolis, GRC; 5 Nursing, Democritus University of Thrace, Alexandroupolis, GRC

**Keywords:** caregiver, cost-effectiveness, costs, dementia, healthcare systems, payment methods

## Abstract

Dementia is a progressive neurodegenerative condition, primarily caused by Alzheimer’s disease, predominantly affecting elderly individuals. Under these circumstances, dementia care focuses on assisting patients with daily living, either in nursing facilities or at home. The condition imposes substantial economic burdens on patients, caregivers, and healthcare systems, particularly in Europe and North America, where precise cost assessments are essential.

This review examines the economic impact of dementia care by integrating diverse cost estimation sources to evaluate the cost-effectiveness of preventive care for high-risk individuals and providing meta-analytic estimates of annual medical, non-medical, and informal care costs per patient, and compares these costs with care effectiveness. The analysis focuses on Europe and the United States, with a greater emphasis on Europe, aiming to encourage further research on preventive strategies and caregiver support.

Dementia presents a significant economic challenge globally, driven by rising healthcare costs and an aging population. The disparities between direct and indirect costs in the US and Europe highlight the impact of healthcare systems and cultural practices on dementia care costs. Preventive measures could significantly reduce long-term treatment costs, making them a crucial investment to alleviate future financial burdens.

## Introduction and background

Dementia is a syndrome with a progressive symptomatology of cognitive and functional impairment. Its most common cause is known to be Alzheimer’s disease; however, other neurodegenerative and cerebrovascular disorders could be a cause of dementia as well [1]. It occurs in patients older than 65 years of age; thus, due to population growth and longer life expectancies, the global number of dementia cases is projected to increase from 55 million in 2019 to 139 million by 2050 [2,3], making this syndrome not only a current issue, but a significant future obstacle as well. Since there is no treatment for dementia, its care consists of assistance and consultation in activities of daily living, either as a community in nursing facilities, or individual care at home [3].

Dementia has a variety of burdens for the patient, the caregiver, and the health care system, its economic aspects being one of the most important ones [4]. Since this is a rapidly increasing issue in Europe and North America, accurate estimates on the costs of care for dementia are gradually more essential [1]. There have been various attempts to estimate the costs of dementia across Europe over the years. For example, it has been estimated that the cost of dementia in Austria in 2009 was approximately 2.9 billion euros [5]. To demonstrate an accurate cost analysis, it would be important to estimate different costs at different stages of dementia severity; however, currently, only a single source of cost estimation data is used [1].

This analysis examines a variety of factors, and the results discussed will reflect these considerations. These factors include the different types of payment methods (public spending, patient/family out-of-pocket (OOP) expenses, or third-party expenditure), the type of services provided (inpatient care, outpatient care, community care/nursing homes, and home-based care), and the types of costs associated with dementia (formal costs/direct expenses and informal costs/opportunity costs borne by caregivers).

The aim of this study focuses on the following issues: i) to investigate the estimated data arising from different sources to assess the cost and provide a cost-effective analysis of the preventive measures that should be considered for individuals at a high risk for dementia; ii) to examine results from a variety of sources associated with a meta-analysis, to calculate the annual medical and/or non-medical as well as the informal care cost, per patient, with the emergence of dementia.

## Review

Materials and methods

**Figure 1:**
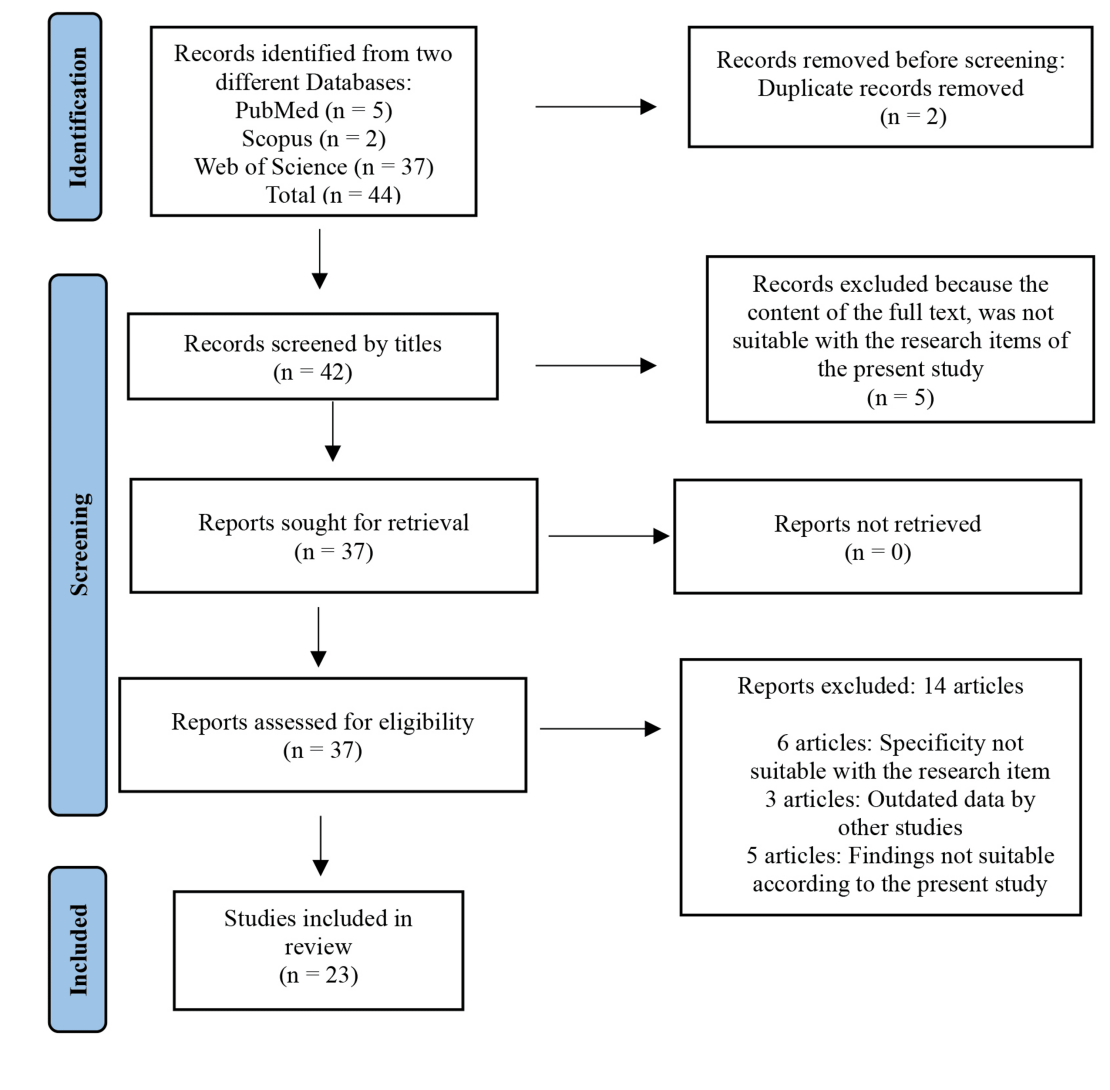
The Preferred Reporting Items for Systematic Reviews and Meta-Analyses (PRISMA) flow diagram demonstrating the selection process of eligible articles

To achieve the objective of this review, a substantial dataset of cost estimation information was gathered from a range of peer-reviewed articles and publications across various scientific sources. Data collection was conducted via scientific search engines, specifically Google Scholar and PubMed, using key terms including "dementia," "preventive care," "cost," "cost-effectiveness analysis," and "Alzheimer’s disease." Only articles published within the last 20 years were included. Figure 1 depicts the selection process of publications used for this review.

This review aims to explore and compare findings from Europe and the United States concerning the costs associated with dementia care. While both regions are analyzed, greater emphasis is placed on Europe. To provide a comprehensive understanding of dementia-related expenses, the discussion will be organized under three overarching scopes [6].

Disease Severity

The first scope addresses how costs evolve as dementia progresses through its various stages. The focus is on the incremental financial burdens associated with disease severity, highlighting how advanced stages lead to significantly higher expenses due to increased care needs, including specialized services and institutional care.

*Source of Funding* The second scope examines the origins of funding for dementia care, categorized into state/public spending, patient/family costs, and third-party expenditure. The first covers the contributions made by government programs or state-funded healthcare systems. The second deals with expenses borne directly by patients and their families. The third pertains to payments made by private insurance providers or other external entities. Notably, attention is given to the interplay of these funding sources in covering dementia care expenses and the degree to which families bear co-payments across different healthcare systems.

*Types of Cost* Under this scope, dementia-related expenses are divided into two categories, namely direct costs and indirect costs. The former covers inpatient care, outpatient care, institutional care, and community-based services such as day care centers or nursing aid at home. The latter is concerning the economic and social burden on informal caregivers, including lost wages, opportunity costs, and physical or emotional stress. Indirect cost is a crucial issue, particularly for caregivers who offer significant support with an impact on their own health status and well-being. Special focus is given to indirect costs, which have recently become a sensitive and significant area of study. Historically underexplored, this dimension sheds light on the socioeconomic challenges faced by caregivers and the broader implications for families and communities.

This review does not present new data but instead aims to synthesize existing research to generate insights and provoke thought regarding dementia care costs in the Western world. By highlighting the disparities and shared challenges between Europe and the United States, this analysis hopes to encourage further research and discussion, particularly around preventive strategies and support mechanisms for families and caregivers [6,7].

Results

The results of this scoping review are categorized into subgroups. Initially, we calculate the mean costs per dementia patient in Europe and the United States, considering various factors. These factors include the severity of dementia symptoms and the types of payers in each country. Specifically, the analysis encompasses costs borne by state/public funding, third-party entities such as private insurers and non-profit organizations, and, lastly, costs incurred by patients, their families, and society at large.

Given the broad and multifaceted nature of the term 'cost,' it is further delineated into subcategories. Direct costs will include expenditures directly incurred by patients, third-party payers, and the state. Indirect costs will address secondary societal burdens, such as the impact on pension systems, taxpayers, and families who may face economic hardship due to caregiving responsibilities, including reduced workforce participation [8,9].

Severity of Dementia

**Figure 2:**
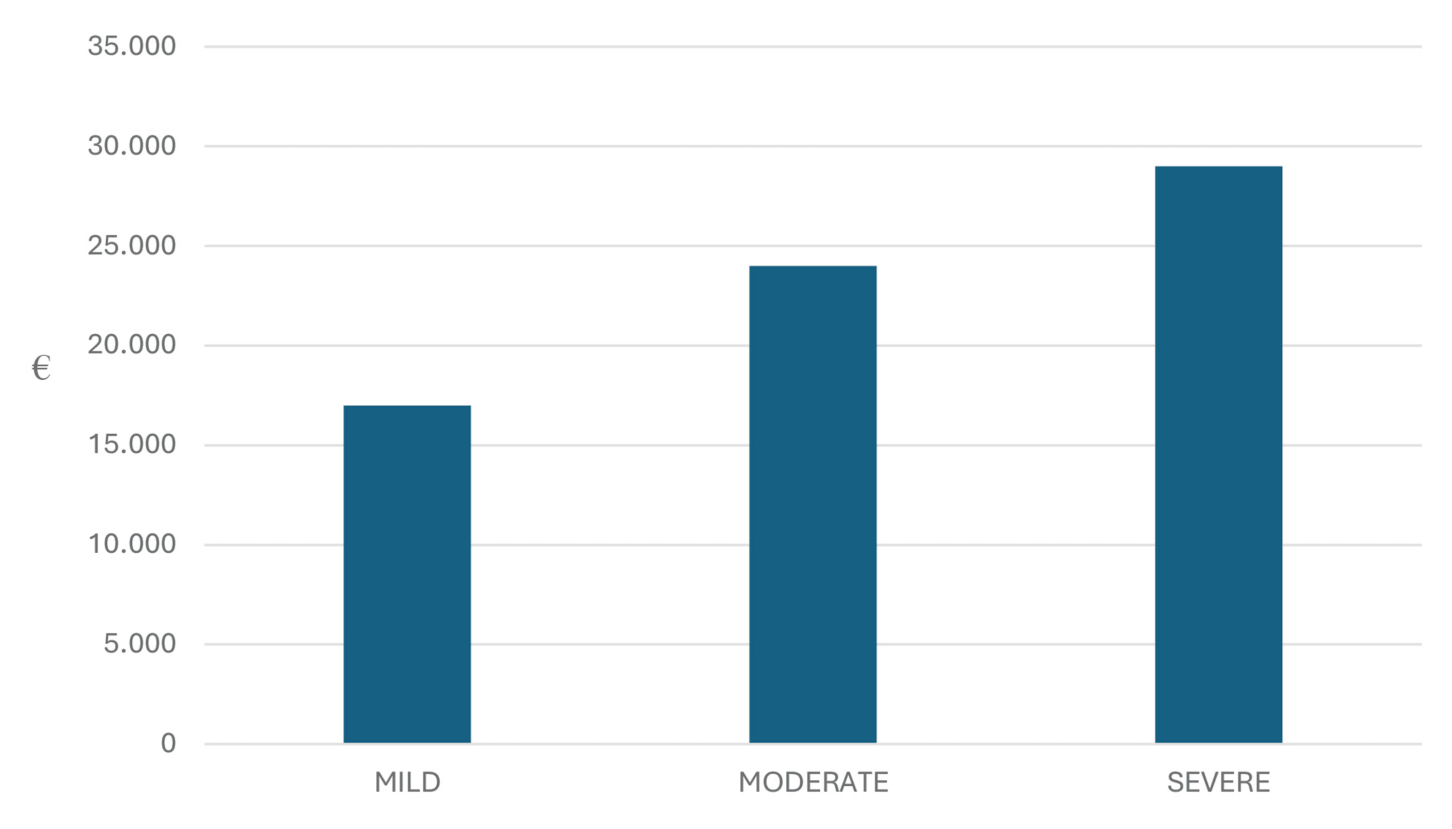
Cost per patient/year by dementia disease severity in Europe 2015 This graph has been created by the authors and features data derived from Braun et al. and Cantarero-Prieto et al. [5,9].

Overall, the differences in the stages of dementia result in varying levels of need for assistive care units, medical devices, personnel, and pharmaceuticals. Consequently, it is logical that costs vary significantly depending on the severity of the disease [5]. In two 2015 studies estimating costs in both Europe and the United States, the more the severity, the greater the cost for both the patient/family and society, with mild dementia costing approximately €17.000 per patient/year in Europe. This cost increases by €7.000 in moderate dementia and by €12.000 in severe dementia (Figure 2) [5,9].

State/Public Expenditure

Healthcare systems are expected to face progressively increasing expenditure rates on dementia care in the near future [4]. From a macroeconomic standpoint, the costs associated with dementia far exceed those of other major diseases currently burdening healthcare systems, including cardiovascular diseases, strokes, cancer, HIV, and diabetes. In some cases, dementia-related costs even surpass those of cancer. This significant financial burden may be attributed to the aging population and the limited availability of preventive care. The reliance on long-term therapies to manage dementia contributes to a financial deficit, which can reach up to 60% [10].

Third Party Expenditure

Third-party expenditure refers to expenses covered by an independent entity on behalf of the patient. This category includes organizations such as private insurance companies and funding programs like Medicare or Medicaid in the United States [11]. A study conducted in Belgium, within the European healthcare context (where programs like Medicare or Medicaid do not apply), included 355 participants comprising both dementia patients and their caregivers. The study revealed that the total mean monthly costs reimbursed from a third-party payer perspective for dementia care amounted to €968 per capita. This expenditure is allocated across various services. One-third of the total costs (€330) is spent on hospital care, while approximately one-third of that amount (€123) is allocated to outpatient visits with a range of specialists, including general practitioners, occupational therapists, psychologists, neurologists, geriatricians, and psychiatrists. Within outpatient care, the largest share, about half, is spent on physiotherapy (€58) [12]. This demonstrates that third-party expenditure is significantly impacted by the costs associated with dementia care.

Patient/Family costs (OOP)

Out-of-pocket spending is a common burden for dementia patients, potentially leading to patients forgoing necessary services and medications or struggling to afford other essential goods. Dementia, due to its progressive nature and association with disability and loss of independence, often exposes patients and their families to high OOP costs, particularly for long-term care services. A study utilizing data from the Aging, Demographics, and Memory Study (a nationally representative subsample (n = 743) of the Health and Retirement Study) analyzed OOP spending among individuals with dementia compared to those with cognitive impairment without dementia and those with normal cognitive function. After adjusting for demographics and comorbidities, the results showed that individuals with dementia had over three times the annual OOP expenses as those with normal cognition ($8,216 for individuals with dementia vs. $2,570 for those with normal cognition). The elevated OOP spending among dementia patients was primarily driven by higher nursing home care expenditures. However, dementia was not associated with an increased likelihood of forgoing prescription medications due to cost [13].

In a different study conducted in the United States with 4.505 participants aged 70 and older found that 1.750 (38.8%) had possible or probable dementia, while 2.755 (61.2%) did not. For individuals with dementia, the median monthly OOP costs for long-term care were $1.465 for those in nursing homes and $2.925 for those in other residential facilities, significantly higher than the $260 for those living in the community. Although these figures were similar to the median OOP expenses for people without dementia in comparable settings, those with dementia faced a much greater risk of incurring catastrophic long-term care costs. The 75th percentile of OOP expenses for dementia patients was $4.566 for those in non-nursing home residential care and $7,500 for those in nursing homes, compared to $3.694 and $3.100, respectively, for those without dementia. Importantly, for individuals with dementia living in care facilities, these median expenses accounted for 100% of their monthly income, underscoring the significant financial strain that dementia care can impose [14].

Direct Costs

Significant variations in dementia-related costs are observed across Europe. A study analyzing cost data from a broad range of sources (n=113) highlights these differences. In Nordic countries, there is a greater reliance on formal care services, in contrast to Southern Europe, where informal care plays a more prominent role. In the British Isles, the cost associated with leisure time loss for caregivers is notably higher compared to other regions [12].

Regarding institutional care, the highest costs are observed in the Nordics, while the lowest costs are found in Southern Europe, followed by the British Isles. Interestingly, the trend reverses in pharmaceutical care, where higher costs are seen in Eastern Europe, the Baltic states, and Southern Europe, and lower costs are observed in the Nordic countries. Western and Eastern Europe, along with the Baltic states, exhibit fewer differences overall; however, both regions show high costs associated with institutional care [12].

Inpatient Care

Calculating the costs of inpatient care exclusively attributable to dementia is inherently challenging. Studies indicate that comorbidities such as type II diabetes, vascular diseases, and depression significantly influence the progression of dementia. In many cases, inpatient care is necessitated by symptoms arising from these comorbidities rather than dementia itself. Without the presence of dementia, the progression and management of these comorbidities might be more favorable, complicating the ability to isolate and calculate costs specifically associated with dementia. This highlights the undeniable link between dementia and its comorbidities, which must be considered when evaluating healthcare costs [15,16,17]

A study conducted by Kumar et al. sought to measure healthcare utilization costs concerning dementia progression. The study compared various metrics, including outpatient visits, emergency department usage, inpatient stays, skilled nursing care, home health services, and total healthcare expenditures, in the years preceding a dementia diagnosis. These metrics were analyzed alongside those of comparable individuals without dementia during a similar stage in life. The findings revealed that healthcare utilization costs for individuals with dementia were nearly double those of their non-dementia peers of the same age and sex. This underscores the substantial financial burden associated with dementia care as compared to the general aging population [18].

Outpatient Care

Outpatient care, such as day care centers, can offer a more cost-effective alternative to standard care. They often include day care centers or outpatient hospital visits with the contribution of many different specialists. A study demonstrated that individuals diagnosed with dementia exhibit significantly higher healthcare service utilization and associated costs compared to their non-dementia counterparts. During a one-year follow-up period, dementia patients displayed notably elevated psychiatric service use, including a seven-fold increase in outpatient visits (2.2 visits compared to 0.3 for non-dementia subjects) and significantly higher costs ($170 vs. $20). Additionally, non-psychiatric service utilization was also greater among dementia patients, with an increased number of outpatient visits (34.4 visits compared to 31.6) and higher total costs ($3827 versus $2389). When examining overall healthcare expenditures, dementia patients incurred total costs that were 1.67 times higher than those without dementia ($3997 versus $2409).

These findings align with prior studies that highlight the substantial economic burden posed by dementia, further reinforcing the significance of this issue in healthcare systems worldwide. The study employed a nationwide, population-based dataset, which minimized selection bias and provided a comprehensive comparison of healthcare service use and expenditures. Despite its robust methodology, the study acknowledged several limitations. The reliance on administrative records for dementia diagnoses may have excluded undiagnosed cases, thereby underestimating the true prevalence and associated costs. Furthermore, the analysis did not account for indirect costs, such as lost wages, long-term caregiving expenses, or the broader societal impacts of dementia [19].

Institutional Care

Systematic reviews have compared hospitalization rates between dementia patients and non-dementia patients, with a focus on whether they were nursing home residents. The findings indicated that nursing home residents with dementia were hospitalized less frequently at the end of life compared to their non-dementia counterparts. However, it is important to note that this review included studies conducted in the United States, Germany, and Canada, countries with varying levels of expenditure on dementia care and healthcare in general. These differences in healthcare systems and spending may influence the generalizability of the findings [20]. 

Community Care

Community care may have a significant positive effect on treatments, in both clinical and economic aspects. Group activities, such as group meditation programs and community-focused treatments, can be obtained by patients in group gatherings, such as day care centers and nursing homes. A study conducted in Germany enrolled 433 individuals with mild dementia or mild cognitive impairment to compare the costs and outcomes of multicomponent, non-pharmacological Motor stimulation, Activities of daily living stimulation, K/cognitive stimulation, Social stimulation (MAKS) treatment vs. 'care as usual' in day-care centers. The study showed that, after six months, the measures used to evaluate the intervention group showed significant improvements compared to the control group. The adjusted cost difference was €938.50. Based on the cost-effectiveness acceptability curve (CEAC), the MAKS treatment was found to be cost-effective for 78.0% of participants on the Mini-Mental Status Examination (MMSE) and 77.4% on the Erlangen Test of Activities of Daily Living in Persons with Mild Dementia or Mild Cognitive Impairment (ETAM), without incurring additional costs to payers [21].

Indirect Costs

The indirect costs considered in this review pertain to the financial and personal burdens faced by family caregivers, including the loss of labor due to caregiving responsibilities, as well as the impact on the quality of life (QoL) for both the dementia patient and the caregiver. Various studies have attempted to measure QoL, which is inherently a subjective variable reliant on individual self-reporting. Demographic factors such as gender, age, and race appear to have limited influence on the QoL of patients as the disease progresses. Although evidence is mixed regarding the relationship between QoL and cognitive decline, neuropsychiatric symptoms, particularly depression, have been consistently observed to increase in severity as the disease advances for caregivers; there is a scarcity of research exploring the progression of their QoL relative to the patient’s cognitive decline. However, the limited evidence available suggests no significant deterioration in caregiver QoL solely due to the progression of cognitive impairment in the patient [22]. In a scope review examining examples of indirect costs through studies from various countries in Europe, notable differences were identified in the total costs of dementia between Europe and the United States. Interestingly, the total costs incurred by dementia patients were found to be higher in the United States (€43,000) compared to Europe (€32,500).

Breaking down these costs, direct medical and non-medical expenses were consistently higher in the United States. Direct medical costs in Europe averaged €11,300, compared to €17,000 in the United States. Similarly, direct non-medical costs, which may include services such as home care or assisted living, were €3,000 in Europe versus €12,300 in the United States. An intriguing finding, however, was the reverse trend observed with indirect costs. Indirect costs, which encompass losses such as reduced productivity or the opportunity costs associated with caregiving, were significantly higher in Europe (€21,200) compared to the United States (€16,600). This divergence underscores the complexity of cost structures in dementia care, influenced by regional differences in healthcare systems, cultural practices, and the reliance on informal caregiving [9].

Caregiver Work Loss

Most dementia patients receive care at home from an informal caregiver, often a spouse or relative, typically female, who assumes the responsibility of providing care. This caregiving role has been shown to have significant impacts on various aspects of the caregiver’s overall well-being. Physically, dementia caregivers often experience challenges such as reduced aerobic endurance, severe back pain, fatigue, and other musculoskeletal disorders. Health issues, including hypertension and cardiovascular diseases, are also prevalent among this population. In addition to these physical and health-related burdens, caregivers face substantial socioeconomic challenges. Caregivers frequently sacrifice their leisure time, social interactions, and financial resources to care for the dementia patient. These sacrifices not only affect the caregiver's economic stability but also contribute to mental health issues, such as subjective burden distress, sleep disturbances, and other psychological disorders [6]. A study conducted by Schwarzkopf et al. utilized administrative data from the German statutory insurance to estimate dementia care expenses. The study revealed that the average annual cost per person with dementia is around €47,561, with over 80% of this cost arising from informal care provided by family members [23].

Discussion

When analyzing data from various sources across Europe and the United States, an intriguing observation emerges. While direct costs per dementia patient per year are notably higher in the United States compared to Europe, indirect costs show the opposite trend, being higher in Europe than in the United States. Despite this reversal in indirect costs, the overall annual expense of dementia care remains significantly higher in the United States. These findings underscore the complex interplay of healthcare infrastructure, cultural practices, and funding mechanisms in shaping the cost dynamics of dementia care. Further research is warranted to delve deeper into these disparities, examining the root causes and potential interventions to optimize resource allocation and reduce the economic burden of dementia on both sides of the Atlantic.

As the disease progresses, the financial burden on all three payer groups increases significantly. Implementing preventive care measures could enable the state, patients, caregivers, and third-party payers to avoid these substantial costs in the future. Therefore, greater emphasis should be placed on dementia prevention rather than exclusively focusing on post-diagnosis treatment. When considering dementia care, preventive measures could play a crucial role in not only slowing the disease's progression but also in reducing the long-term treatment costs by up to 60%. This highlights the importance of investing more in preventive care, as it could alleviate the financial strain on healthcare systems in the future.

The observed disparity in dementia-related costs between regions could potentially be attributed to differences in healthcare systems and caregiving practices. For instance, in the United States, the absence of comprehensive state-funded care often results in greater reliance on private insurance and OOP expenditures, which significantly increase direct costs. In contrast, Europe exhibits a greater prevalence of informal caregiving, typically provided by family members or close relatives. While this reduces direct costs, it leads to higher indirect costs due to factors such as lost wages, opportunity costs, and the broader socioeconomic burden borne by caregivers.

This distinction underscores the importance of tailoring dementia care strategies to the unique characteristics of healthcare systems and cultural practices in different regions. The reliance on informal care in Europe highlights the need for policies that support caregivers, such as wage replacement programs or caregiver training. Conversely, the financial pressures associated with private expenditures in the United States suggest a potential benefit from expanding access to state-funded or subsidized care options.

Furthermore, additional research is needed to evaluate the potential advantages of preventive care as a cost-effective alternative to predominantly treatment-focused approaches. Preventive strategies may alleviate the financial burden on healthcare systems, patients, and caregivers by reducing the incidence or delaying the onset of dementia. Without timely intervention, the availability and quality of preventive care could deteriorate, further exacerbating the economic and social challenges posed by dementia. This is particularly crucial as the global population continues to age and the costs associated with dementia care are projected to rise.

## Conclusions

Dementia represents a substantial economic challenge for countries across the globe. As populations continue to age and healthcare costs steadily rise, the financial burden of managing dementia care has become an increasingly pressing issue for many healthcare systems. In terms of funding dementia-related healthcare, the largest share of costs is typically borne by OOP expenses and public expenditure, with third-party payments accounting for a relatively smaller portion. It is essential to note that, based on the reviews referenced in this overview, both third-party expenditure and public spending in all instances involved some level of patient contribution through co-payments. The amount and percentage of these co-payments vary significantly across different countries and healthcare systems, reflecting the diversity in health financing models. Therefore, to address these disparities in costs, it is essential to prioritize the development and implementation of effective preventive strategies, ensuring that healthcare systems worldwide are better equipped to manage the growing burden of dementia sustainably and equitably.
